# General practitioners’ management of depression symptoms in Somali refugee and Norwegian patients: a film vignette experiment

**DOI:** 10.1136/bmjopen-2021-055261

**Published:** 2021-12-28

**Authors:** Samantha Marie Harris, Per-Einar Binder, Esperanza Diaz, Vebjørn Ekroll, Gro M Sandal

**Affiliations:** 1 Department of Psychosocial Science, University of Bergen Faculty of Psychology, Bergen, Norway; 2 Department of Clinical Psychology, University of Bergen Faculty of Psychology, Bergen, Norway; 3 Department of Global Public Health and Primary Care, University of Bergen Faculty of Medicine and Dentistry, Bergen, Norway; 4 Unit for Migration and Health, Norwegian Institute of Public Health, Oslo, Norway

**Keywords:** mental health, primary care, public health

## Abstract

**Objectives:**

Refugees in comparison with non-refugee patients may face higher unmet mental healthcare needs. The mechanisms underlying these disparities are still poorly understood. The general practitioner (GP) plays a vital role in refugees’ mental health (MH), managing complaints within primary care and acting as gatekeeper to specialist services. However, GPs have reported feeling uncertain about working with refugee patients. Somalis make up one of the largest refugee groups in Norway and use primary care services more than the majority population for physical health, although not for MH. The current study examines GPs’ management of MH complaints in Somali refugee versus Norwegian vignette characters and the role of GP clinical uncertainty.

**Design:**

We distributed an online experimental survey to GPs in Norway (n=133), who were randomised to watch a simulated consultation with a female Norwegian, female Somali, male Norwegian or male Somali vignette character, presenting the same symptoms of depression. GPs indicated which diagnoses, assessments and treatments they would endorse for the patient and their level of certainty.

**Outcome measures:**

We calculated Simpson indices to measure inter-rater reliability and 2×2 analysis of variances as well as Bayesian estimation to examine clinical certainty based on patient background and gender.

**Results:**

GPs’ clinical decisions about Somali and Norwegian vignette characters were similar, with a few exceptions. There was less consensus regarding the first prioritised diagnosis for Somali characters (Simpson index=0.129) versus Norwegian characters (Simpson index=0.208), (p=0.011, one-tailed). Somalis more frequently received diagnoses of post-traumatic stress disorder (PTSD), while Norwegians received diagnoses of feeling depressed. GPs endorsed sick leave more often for Norwegian characters and medication for physical complaints for Somali characters. There were no substantial differences in GPs’ self-reported certainty.

**Conclusions:**

We found few and relatively small effects of patient background and gender on GPs’ clinical decisions. Nevertheless, the validity of certain diagnoses and prescription of sick leave need to be considered by clinicians and in future research.

**Strengths and limitations of this study**

- The experimental design has limited external validity and may not translate directly to a true consultation setting.
- Findings may be less relevant regarding clinical decisions made by other health professionals, or clinical decisions made about patient populations other than Somali refugees.
- However, the experimental approach provides practically unconfounded comparisons of vignette characters’ gender and background.
- By specifying the patient’s background, we were able to improve the relevance of the findings for general practitioners and service users with a Somali background.
- The power analysis followed by both frequentist analyses as well as Bayesian estimation provides a statistically robust basis on which to draw the conclusions.

## Introduction

Since the beginning of the so called ‘refugee crisis’ in 2015, much attention has been paid to delineating the difficulties refugee populations face regarding health and healthcare.^1–5^ Previous literature from European countries suggests that there are persistent inequalities between refugee/migrant and non-migrant groups, with migrants facing higher levels of unmet healthcare needs particularly regarding mental health (MH).^4^ However, the underlying mechanisms of these disparities are still poorly understood.

The general practitioner (GP) plays an important role in refugees’ MH care, resolving most MH problems within general practice as well as acting as gatekeeper to secondary services. The Norwegian public healthcare system is characterised by universal health coverage for all legal residents, including refugees, and individuals make only modest copayments of maximum 2460NOK (€247) annually for different services. Services covered by universal healthcare include primary care, hospital care, and mental healthcare. As a result, all residents are encouraged to seek help from their GP for both physical and MH problems.^6^

There are currently more than 27 000 (number retrieved by internal employee from table "08144: Personer med flyktningbakgrunn, etter statistikkvariabel, flyktningstatus, år, region og landbakgrunn") Somali refugees in Norway (Statistics Norway, personal communication, 1 October 2021). Refugee groups, in general, have reported poorer MH,^1 2 5 7–10^ and lower use of health services than the majority population.^11 12^ However, this pattern may not apply to Somali refugees, who have self-reported good physical and MH,^13 14^ are more likely to make use of GP services than other sub-Saharan migrants in Norway for somatic complaints,^15^ and have higher contact rates to emergency services than the majority population.^16^ Despite higher contact rates regarding somatic health complaints, Somali women in particular may have lower use of secondary MH services.^17^ This pattern does not necessarily mean that they do not experience psychological distress, however.^18^ Lower contact rates to MH services may be the result of high levels of stigma attached to mental illness,^19^ the belief that MH problems do not exist among Somalis^20^, and that mental illness ought to be treated through spiritual approaches.^21^ Additionally, lower contact rates to specialist MH services may reflect lower referrals from GPs.^22^

A United Nations High Commissioner for Refugees (UNHCR) report on the culture, context, and MH of Somali refugees has suggested that health professionals find it challenging to provide healthcare to Somalis with a refugee background suffering from MH problems, due to their psychosocial problems and distinct cultural and religious conceptualisations of MH,^19^ which may include the belief that mental illness is the result of spiritual possession or of being a bad Muslim.^20 21^ While the knowledge that a patient has undergone forced migration may increase empathy towards refugees among host country residents, it may also increase anxiety and feelings of threat.^23^ GPs may, therefore, experience distinctly different psychological responses to refugee patients in comparison with non-refugee patients. This is supported by findings indicating that health professionals evaluate forcibly displaced patients differently from majority population and other patients with foreign descent, for example being less optimistic about their recovery^24^ and feeling less confident about providing care to them.^25–28^ Similarly, Somali women with a refugee background may be perceived as being more at risk for violence,^29^ such as female genital mutilation,^30^ which may influence GPs’ clinical decisions about this patient group. The role of gender in clinical decisions about diagnoses and treatments has been previously documented among non-migrant patients,^31–33^ and it has been shown that migrant women, compared with men, may receive fewer follow-ups for common mental disorders in Norway.^34^ However, less is known about gender differences in the management of MH problems among individuals with a refugee background.

There is evidence suggesting that some migrant groups may be handled or managed differently in primary care. Somali and Iraqi migrants, for example, have been referred more often for laboratory tests for physical health complaints in emergency primary healthcare services for non-urgent purposes, in comparison with German and Polish migrants, as well as the majority population in Norway.^16^ Whether this is due to a real difference in their need for laboratory tests is unclear. Still, it is noteworthy that the overuse of diagnostic tests has previously been identified as an indicator of clinical uncertainty on the part of the practitioner.^35 36^ In the UK, it was found that the apprehension some GPs experienced in working with individuals from other ‘ethnicities’ (the use of this term is not further clarified in the article) could be debilitating to their practice.^28^ Improved cultural competence has been shown to improve health professionals ‘transcultural self-efficacy’, or the confidence with which they approach intercultural clinical consultations.^37^ Furthermore, cultural competence contributes to health professionals attributing more trustworthiness to asylum-seeking patients.^24^ Feeling a lack of cultural competence, for example through lacking training or courses, may therefore play a role in the experience of clinical uncertainty in inter-cultural consultations.

Clinical uncertainty is inherent to clinical practice,^38^ but may be exacerbated in consultations with vulnerable patient groups and is further ‘complicated by cultural differences and psychological challenges’.^39^ GPs have previously reported mismatched expectations of treatment as well as different understandings of MH, as particularly pertinent barriers to providing effective mental healthcare to refugee patients.^27 40–42^ These barriers may furthermore contribute to GPs’ feelings of being unprepared and uncertain about their clinical decisions.^27^ This is supported by evidence that GPs in the UK and USA experienced greater clinical uncertainty diagnosing depression among African Caribbean and African American individuals respectively.^43^ Clinical uncertainty is particularly relevant in primary care consultations, where clinicians are often confronted with undifferentiated illness presentations, and in MH consultations.^38 44^ Uncertainty can exist at the individual level as well as the aggregate level, which has been referred to as microuncertainty and macrouncertainty, respectively.^45^ Microuncertainty refers to self-reports of clinicians’ uncertainty, while macrouncertainty refers to a lack of consensus across clinicians.^45^ However, medical curricula and the culture of medicine place little weight on acknowledging, accepting and managing uncertainty in a clinical context.^38 46^

Since uncertainty is unavoidable in a clinical consultation the ‘key dilemma’^47^ is how clinicians make decisions when faced with the reality of uncertain situations. When faced with situations with insufficient information and high uncertainty, health professionals are likely to rely on heuristics when making decisions.^48^ In a clinical setting, this means that a clinician may rely on what they assume about, for example, the patient’s background and gender, to conclude which diagnoses, assessments and treatments are most appropriate. This type of decision-making has the advantage of being efficient, however, it is also prone to human error and bias.^48^ The risks of clinical uncertainty, therefore, include higher variation in the clinical decisions made about different patients, and ultimately, disparities in treatment offered, even when individuals present with comparable complaints.

Studies that present the disparities between migrant and majority populations are typically observational and cannot fully consider potentially confounding variables.^49 50^ Experimental approaches may be the only feasible way to examine the unconfounded impact of patient characteristics on clinicians’ decisions.^51^ In the present study, we examine differences in the diagnoses, assessment and treatment options chosen by GPs regarding female and male Norwegian and Somali refugee vignette characters presenting with the same symptoms of depression in simulated primary care consultations. We predicted that there would be less consensus among GPs (higher macrouncertainty) regarding the most appropriate diagnoses, assessments and treatments considered for the Somali group versus the Norwegian group. Furthermore, we hypothesised that participants would self-report lower rates of certainty (higher microuncertainty) in consultations with the Somali characters. Finally, we hypothesised that we would observe an interaction of patient gender and background regarding both macrouncertainty and microuncertainty.

## Methods and materials

### Sampling and recruitment

The sample included 133 individuals currently working, or having previously worked, as GPs in Norway. Participants were recruited through snowball sampling, and via Bergen municipality’s newsletter, through convenience sampling from August to November 2020.

We conducted a power analysis^52^ for the 2×2 analysis of variance (ANOVA) examining GPs’ self-reported certainty (dependent variable) based on background and gender of the patient (independent variable), which indicated a required sample of 128 participants in total to achieve a power of 0.80 for detecting a medium effect size of *f*=0.25 at a 5% alpha error level. We chose an effect size of 0.25, because we felt that smaller effects were unlikely to have a large practical consequence, although it must be kept it mind that also small effect sizes may have implications on a population level. A total of 192 participants took part in the study, however only 137 completed the entire survey. Participants who did not meet the inclusion criteria, that is, had never worked as GPs, were excluded (n=4). The remaining 133 participants were included in the final analyses. A Consolidated Standards of Reporting Trials flow diagram can be found in online supplemental figure S1 of the supplementary material. Participant characteristics are presented in online supplemental table S1 of the supplementary material.

10.1136/bmjopen-2021-055261.supp1 Supplementary data

**Figure 1:**
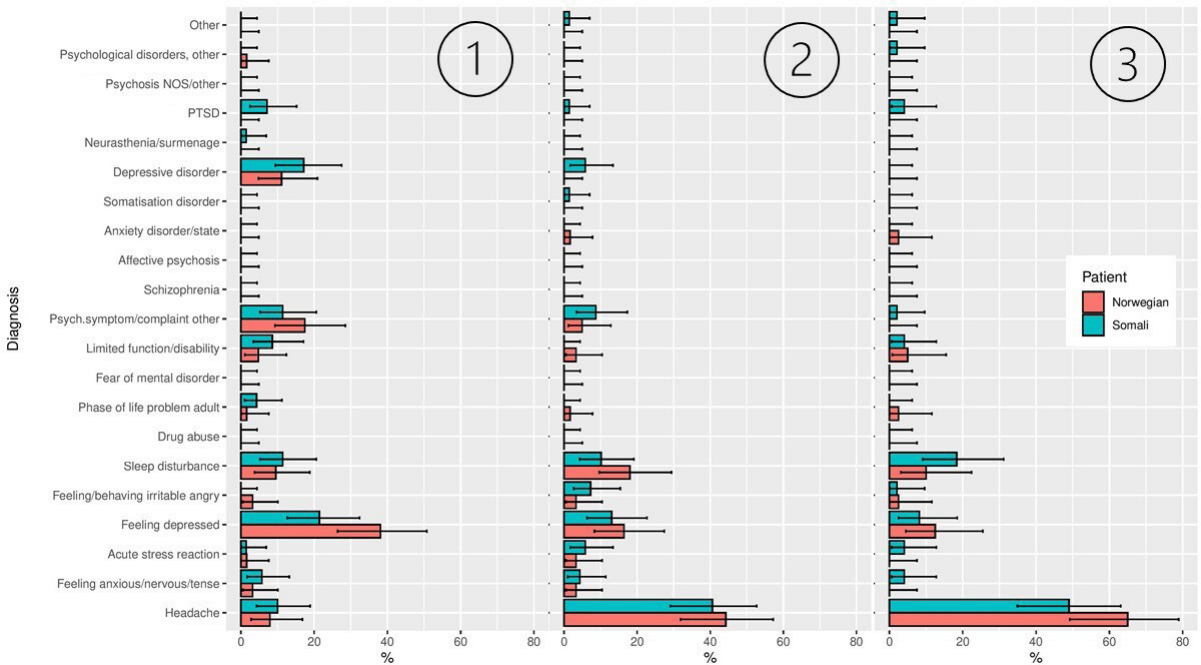
Distributions of diagnoses chosen, plotted separately for Norwegian and Somali vignette characters. First, second and third ranked priorities are displayed from left to right. The error bars show 95% HDI of the posterior based on a uniform prior. HDI, highest density intervals. NOS, not otherwise specified.

Eighty participants (60.2%) reported either not having taken, or not remembering having taken, a course on migration and health during their education, and 97 (72.9%) gave the same answer regarding such a course after finishing their education. However, 89 participants (66.9%) indicated that they felt the need for a course in migration and health.

### Procedure

The survey was distributed online via the survey platform Survey Xact.^53^ Using simple randomisation, participants were allocated to one of four groups within the survey, each watching one of four film vignettes depicting a simulated primary care consultation with a single character who was either a Somali male (‘Abdi Warsame’), Norwegian male (‘Emil Olsen’), Somali female (‘Hodan Osman’) or Norwegian female (‘Mari Berg’).

Participants indicated up to three possible diagnoses, assessments and treatments they would endorse for the vignette character and ranked the options according to priority. These will be referred to as D1, D2, D3, A1, A2, A3, T1, T2 and T3, where the letters are short for diagnosis, assessment and treatment, respectively, and the numbers represent first, second, and third priority. Participants also indicated their level of certainty in each of these clinical decisions on an 8-point Likert scale where 0 = ‘very uncertain’ and 7 = ‘very certain’. The diagnostic options were based on the International Classification of Primary Care codes.^54^ The assessment and treatment options were developed by the authors, of whom ED is a medical doctor and specialist in Family Medicine.

### Film vignettes

Symptoms presented by the vignette characters were based on diagnostic and statistical manual of mental disorders (DSM-V) and ICD-10 criteria for depression.^55 56^ However, similar to Lawton *et al*’s study,^57^ symptoms were presented in a relatively ambiguous manner so that a range of diagnoses, assessment, and treatment options could be considered appropriate. To improve the realism of the scripts, we included small gender differences in the expression of symptoms based on previous literature.^58^ Consequently, the female vignettes highlighted feelings of depression more in line with diagnostic criteria, including guilt and self-blame, while the male vignettes highlighted being in a bad mood and feeling irritable. Authors SMH, P-EB and GMS attended all film sessions to ensure the same symptoms were presented in the same order and that the actors’ body language was comparable.

The vignettes were filmed at Media City Bergen. Actors (Figure S2) were recruited from the Bergen amateur dramatic society and the research group’s network. All actors were fluent in Norwegian. However, the Somali actors both spoke with a Somali accent, making similar, small linguistic mistakes in Norwegian. The actors’ ages ranged from 26 to 32. The GP character, who was briefly visible in all four clips, was played by the same actor. Each participant received background information about the vignette character before the consultation (Box S1).

**Figure 2:**
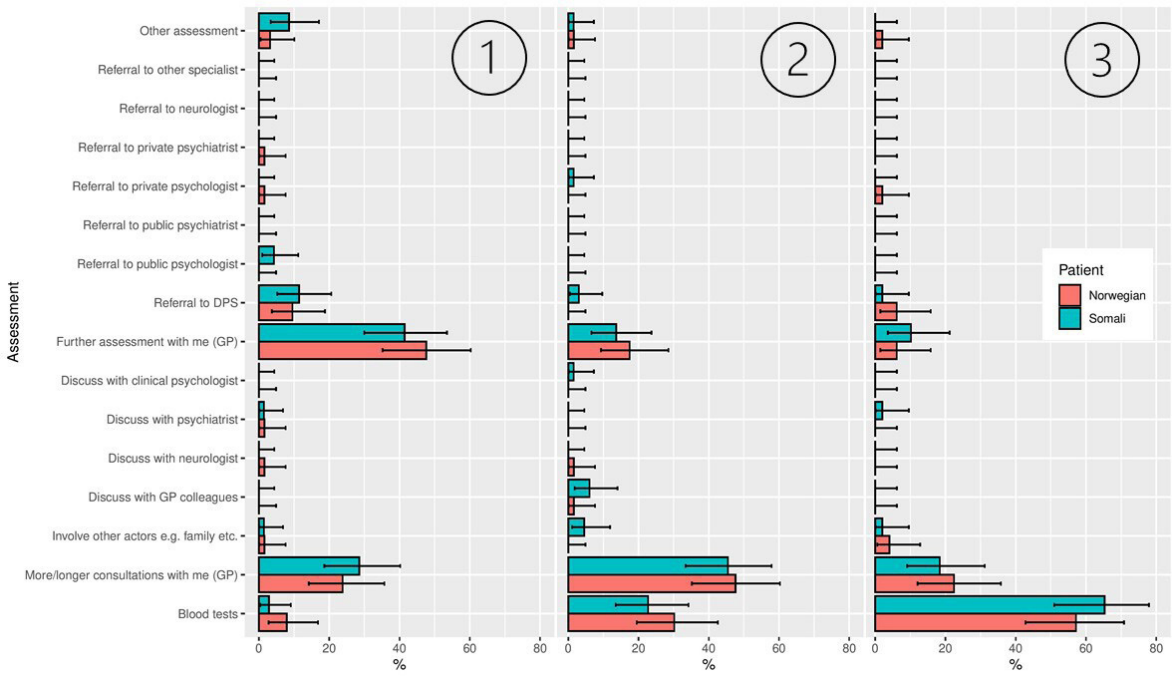
Distributions of assessment options chosen, displayed by vignette character background. First, second and third ranked priority are displayed from left to right. The error bars show 95% HDIs of the posterior based on a uniform prior. Note that ‘Referral to DPS’ refers to referrals to the regional psychiatric centre/community mental health services (in Norwegian: distriktpsykiatrisk senter). These centres are part of secondary services and provide specialist psychiatric care. HDI, highest density intervals.

### Patient and public involvement

The scripts were developed by authors SMH, P-EB, ED and GMS, as well as two medical doctors from a reference group of stakeholders, of which one has a Somali background. The survey was piloted among 12 medical students, who suggested to include information that the patient’s symptoms had no known somatic causes.

### Statistical analyses

Main analyses were preregistered. Preregistration, data and materials are publicly available on the Open Science Framework (osf.io/qexrj).^59^

#### Simpson indices

As a measure of inter-rater agreement with respect to the diagnosis, assessment, and treatment options chosen by the participants, we calculated the Simpson indices of the distributions of corresponding responses. If the distribution of responses in any given task (say, D1) is 100% for one of the response options and 0% for all others, the inter-rater agreement may be considered perfect. In that case, the Simpson index, which is defined as

$$S=\sum_{i}^{K}{p_{i}}^{2},$$

where *p_i_* is the relative frequency of responses for response option *i* and *K* is the number of available response options, is 1. If the distribution is entirely flat, however (ie, *p_i_=*1 /K), one may say that there is no inter-rater agreement. In this case, the Simpson index equals 1 /K. Hence, the Simpson index can be considered a measure of inter-rater agreement that ranges from 1 /K (minimal agreement) to 1 (maximal agreement). Couched in terms of probabilities, it can be shown that the Simpson index represents the probability that two randomly chosen responses sampled from the theoretical probability distribution are identical.

To test for statistically significant differences between the Simpson indices for different groups, we used bootstrapping^60^ since the theoretical distribution of the Simpson index under the null hypothesis is unknown. More specifically, we used the percentile bootstrap and one-tailed tests.

#### Frequentist ANOVA

To examine whether GPs’ self-reported certainty about their clinical decisions depends on the background and gender of the vignette characters, as well as any interaction of these two factors, we conducted 2×2 independent samples ANOVAs.

### Bayesian estimation

In addition to the frequentist ANOVAs, we used corresponding Bayesian estimation,^61^ which provides richer information, including distributions of credible values for the means and differences of means. Note that credible intervals, 95% highest density intervals (HDI) in Bayesian analysis, in contrast to traditional CIs, indicate the range within which our true value is most likely to lie. We performed the estimation using the hierarchical model (as well as the accompanying R-script) described in Kruschke,^61^ which uses vague priors for the main and interaction deflections. The model assumes that the standard deviation (SD) of the error is normally distributed and equal in all groups, and the prior used for this parameter was a uniform distribution ranging from 0.01 to 10 times the SD in the data. For the overall level across all groups, the prior was a normal distribution centred at the grand mean with a SD five times the SD in the data. The deflections from the overall level corresponding to each of the two factors, as well as the interaction deflections were assumed to be normally distributed and centred at zero, and the prior for the corresponding SD was a gamma distribution with a mode corresponding to half of the SD in the data and a SD twice that of the data.

All analyses were conducted using R.^62^ The Bayesian ANOVA was conducted following the procedures outlined in Kruschke,^61^ using JAGS V.4.3.0.^63^

### Missing data

Participants were asked to rank clinical decisions, indicating *up to* three choices, which led to missing data in the second and third rankings. While the second ranked choices still fulfil the required number of observations for the above-mentioned power analysis (N>128), the third ranked choices, although they were included in the analysis, no longer have the power required to identify an effect of *f*>0.25 (N=89 for D3, N=98 for A3, and N=74 for T3).

## Results

### Clinical decisions and macro-uncertainty

Figure 1 shows the distributions of the diagnoses assigned to the Somali and Norwegian vignette characters.

Somali characters were the only group to receive a diagnosis of ‘P82 PTSD’ for D1. The Norwegian characters were more often given a diagnosis of ‘P03 Feeling depressed’ than the Somali group for D1. There was no strong statistical evidence of a difference in diagnoses chosen for D2 and D3.

Simpson indices and p-values are reported in table 1. As can be seen in figure 1, the Somali group had a broader spread, that is, flatter distribution, than the Norwegian group, indicating less consensus across GPs regarding the most appropriate D1 for the Somali characters. No statistically significant difference regarding the Simpson indices was found for D2 or D3.

**Table 1:** Simpson indices and p-values for ranked diagnoses, assessment, and treatment options for Somali vs Norwegian groups

Clinical decision	Somali	Norwegian	P value
D1	0.129	0.208	0.011
D2	0.214	0.263	0.193
D3	0.289	0.453	0.052
A1	0.277	0.301	0.334
A2	0.284	0.349	0.117
A3	0.472	0.387	0.810
T1	0.183	0.213	0.146
T2	0.298	0.313	0.291
T3	0.456	0.325	0.905

A higher Simpson index indicates higher consensus.

Figure 2 shows the distributions of the assessments assigned to the Somali and Norwegian vignette characters. We found no statistically significant differences between assessment options chosen or Simpson indices (table 1) regarding A1, A2, or A3.

**Figure 3:**
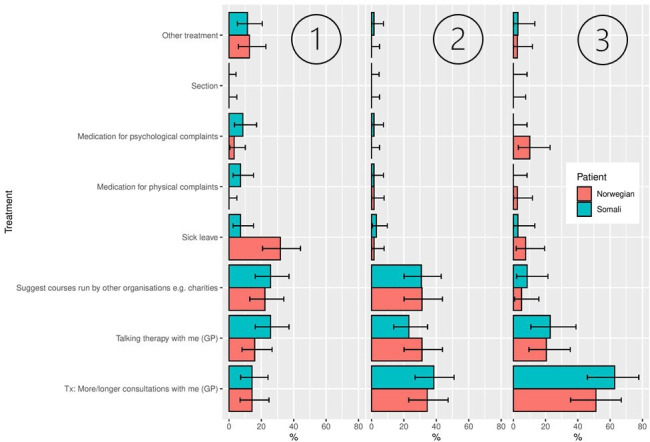
Distributions of treatment options chosen, displayed by vignette character background. First, second and third ranked priority are displayed from left to right. The error bars show 95% HDIs of the posterior based on a uniform prior. HDI, highest density intervals.

**Figure 4:**
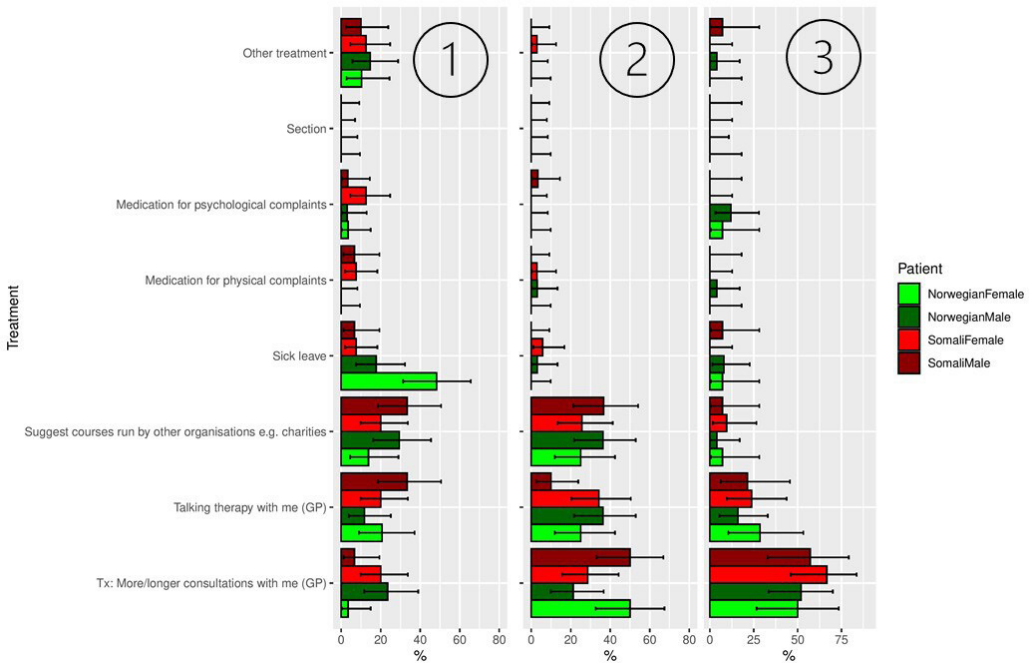
Distributions of treatment options chosen, displayed by vignette character. First, second and third ranked priority are displayed from left to right. The error bars show 95% HDIs of the posteriors based on a uniform prior. HDI, highest density intervals.

The corresponding distributions of treatment options are shown in figure 3. Somali characters were more often prescribed medication for physical complaints than Norwegian characters, while Norwegian characters were given more sick leave (figure 3). However, when examining the four vignette characters separately (figure 4), it appears that the female, Norwegian vignette character received the highest frequency of sick leave in comparison with the other three vignette characters, and may, therefore, account for the difference between the Norwegian and the Somali group in figure 3.

There was no strong evidence for differences between the Simpson indices for the Somali and Norwegian vignette characters regarding T1, T2, or T3 (table 1).

We did not find any statistically significant interaction effect of vignette character gender and background regarding Simpson indices, that is, macrouncertainty, of clinical decisions made by GPs.

There was no statistical evidence that GPs perceived the severity (1=not severe, 4=very severe) of the Somali vignette characters (M=2.86, SD=0.35) symptoms differently from that of the Norwegian ones (M=2.92, SD=0.37), (*t*(128)=1.01, p=0.316).

### Microcertainty

The frequentist ANOVA suggested that GPs’ mean certainty ratings did not differ between the Somali and Norwegian vignettes for diagnoses and assessments. We found, however, that GPs’ mean certainty ratings for T1 were lower for the Somali characters (M=5.9, SD=1.3) than for the Norwegian ones (M=6.3, SD=0.9). While this difference is statistically significant (*F*(1,129) = 4.318, p=0.040), it is small (0.39) and can be considered as having limited practical consequence. We did not find any interaction of the patient gender and migrant background regarding GPs’ self-reported certainty ratings (online supplemental table S2).

We also used corresponding Bayesian estimation to examine posterior distributions (the posterior probability of a given event is the updated probability assigned after new data are taken into account) of the differences in GPs’ self-reported uncertainty regarding clinical decisions made about the Somali versus the Norwegian characters as well as examining the interaction of patient gender and migrant background. In all cases, except T1, a zero difference fell within the 95% HDI. All modes of the posteriors only deviated from zero by amounts smaller than one unit, including that for T1 (Mode=0.387). Hence, any real differences larger than one unit on the 8-point certainty scale are implausible in all cases. Furthermore, findings suggested that any interaction of patient gender and background was likely to have been small.

## Discussion

Overall, the findings suggest that clinical decisions made by GPs about the Somali and the Norwegian vignette characters presenting symptoms of depression were similar, with a few differences found. The findings furthermore suggest that any clinical variation observed is unlikely to be due to differences in GPs’ self-rated clinical uncertainty.

We found a slightly larger spread in the first ranked diagnosis considered for the Somali versus Norwegian vignette characters (ie, higher macrouncertainty). This suggests that there was less consensus among GPs regarding which diagnosis (ranked first priority) was most suitable for the Somali versus the Norwegian characters. Despite having found higher macrouncertainty regarding the first ranked diagnosis for the Somali versus the Norwegian characters, we did not find differences in microcertainty, meaning GPs reported feeling similarly certain about the diagnoses they chose for the Somali versus the Norwegian characters despite different degrees of consensus across GPs. We did not find less GP consensus regarding assessment or treatment options chosen for the Somali versus the Norwegian vignette characters. We did, however, find differences in the type of treatment options that were ranked first for both groups. The most striking of these differences was the relatively high frequency of participants that endorsed sick leave as a treatment for the Norwegian versus the Somali vignette characters. We discovered that the Norwegian female vignette character was more often given sick leave in comparison with the other three characters. This is in line with findings showing that women are more likely to receive sick leave than men in cases of depression.^31^ However, this was not the case for the Somali, female vignette character, despite all vignette characters having explicitly stated that they are employed. Sick leave prescription has previously been associated with a patient’s ability to evoke sympathy.^64^ This begs the question whether the female Norwegian character evoked more sympathy among GPs than the Somali female and the male vignette characters.

GPs also considered medical treatment for physical complaints more often for the Somali characters versus the Norwegian characters. Finally, in contrast to our hypotheses, we did not find that GPs reported higher uncertainty for any clinical decisions, apart from first ranked treatment, and this difference was very small. Nor was there any strong indication of an interaction of patient gender and background regarding certainty ratings. This was further supported by Bayesian estimation.

The higher frequency of PTSD diagnoses for the Somali characters is in line with findings from a previous vignette study.^22^ According to the DSM-5,^55^ symptoms that are indicative of PTSD include negative alterations in cognitions and mood, negative emotional states, diminished interest in activities and detachment from others. These symptoms have substantial overlap with depressive symptoms and could, in combination with the belief that refugees are likely to have been exposed to actual or threatened death, injury or sexual violence, have led GPs to choose the PTSD diagnosis for the Somali refugee versus the Norwegian vignette characters. However, these criteria should not usually be sufficient to meet a PTSD diagnosis, which in addition to exposure to threat and negative alterations in cognition and mood must include intrusion symptoms associated with the traumatic event and persistent avoidance of stimuli associated with the traumatic event. It is possible that GPs tentatively set the PTSD diagnosis with the intention to further investigate whether these remaining symptoms of PTSD are present. This pattern may also suggest the presence of heuristic decision making on the part of the GP. Previous research has consistently found a higher prevalence of PTSD among refugees than among majority populations as well as non-refugee migrants.^65–67^ This knowledge, as well as knowledge about the types of perilous events that refugees encounter,^23^ may have informed GPs’ diagnostic decisions. While it has been argued that doctors *should* be influenced by their knowledge of refugee health when making clinical decisions,^51^ this also leads to the concerning conclusion that some epidemiological data regarding the prevalence of PTSD among refugees may be based on diagnoses that are given despite insufficient evidence of PTSD symptoms. Since observational studies^65–67^ cannot take into consideration the impact patient characteristics may have had on health professionals’ clinical decisions, our findings make an important contribution and indicate that the validity of PTSD diagnoses should receive specific focus in future research. Furthermore, health professionals need to be aware of the risk of overdiagnosing and misdiagnosing clinical mental disorders among refugees based on their own expectations of this patient group.^2 23^

Our findings also partly mirror the macrouncertainty/microcertainty phenomenon, originally observed by Baumann and colleagues,^45^ who described the relative self-reported clinical certainty despite a lack of consensus across health professionals. They concluded that this phenomenon indicates the presence of overconfidence. In a genuine clinical setting, overconfidence may lead to an unwillingness to take in new, conflicting evidence and instead leads to focusing on information that confirms one’s original hypothesis.^45^ This may contribute to an illusion of certainty, in that each GP feels relatively certain of his or her clinical decision, despite a lack of consensus across GPs. However, it is important to note that we did not find a large conflict of macrocertainty and microcertainty. Nevertheless, GPs should remain open to conflicting evidence that may alter what diagnosis, and hence treatment, they believe is most suitable for the patient.

Finally, we found that GPs more often endorsed physical health medication for Somali characters versus Norwegian characters. This may be the result of GPs’ expectations that patients with a refugee background are less open to discussing MH, and therefore more sceptical of MH treatment, and/or may result from the expectation that refugee patients tend to want a ‘quick fix’.^27^ It may also be an indication of GPs’ self-perceived poor cultural competence. This is indicated by the majority of participants (66.9%), who claimed they would benefit from a course in migration and health. As a result, the choice may indicate a premature closure, which refers to the tendency to stop inquiring once a possible solution for a problem is found.^38 68^ This may occur when practitioners are confronted with data they find difficult to interpret or synthesise and make a clinical decision in order to reduce unpleasant psychological tension.^38^

### Limitations and strengths

The current study should be considered in light of the following limitations. While the experimental design of our study has several strengths, it also has limited external validity and may not translate directly to a true consultation setting, where patients and doctors are likely to enter a negotiation influencing the clinical decisions. While the Somali vignette characters had authentic Somali accents and made small linguistic mistakes in Norwegian, we were unable to simulate true language and cultural barriers as this would have made comparison between vignette characters impossible and jeopardised the experimental design of the study. Next, we distributed a link to the survey through various channels. We were only able to register the number of respondents who opened the survey, without knowing how many people had received a link to the survey. Consequently, we were unable to ascertain the response rate. Additionally, convenience sampling and the inclusion of inactive GPs, may have biased the representativeness of the sample. Social desirability may also have been a limitation. Participants were informed that the survey aimed to gather information regarding GPs’ experiences providing healthcare to different patient groups. Although we did not mention that this referred specifically to patients with a migrant background, participants particularly in the Somali vignette character group may have understood the purpose of the study and adjusted their answers to avoid seeming prejudiced, although it is unclear in what way this may have biased the results. However, due to the anonymity of the survey, social desirability may have been less of a risk than in interview studies for example.^69^ Considering that GPs were our target group, findings should be interpreted with caution regarding the clinical decisions of other health professionals, such as psychologists. However, there are certain parallels between the experiences of psychologists and GPs working with refugee patients.^27 70^ For example, it has been suggested that psychologists too report feeling somewhat poorly prepared to work with this patient group.^27 70^ Psychologists’ clinical decisions in sessions with refugee patients should, therefore, be examined in future research. Similarly, our findings may not be as relevant regarding clinical decisions made about other refugee/migrant populations. Findings may, furthermore, have been more useful and generalisable with the use of several vignettes, ideally with patients of different foreign descent, per participant. Finally, while the Somali vignette characters were presented as refugees, we did not clarify the difference between a refugee, asylum-seeker, and undocumented migrant. While we assumed that GPs were aware of the difference, different understandings of the legal rights of refugees may have influenced GPs clinical decisions.

The study also has the following strengths. The experimental approach we used provides practically unconfounded comparisons of vignette characters’ gender and background.^51^ Despite limitations of external validity, our focus on making sure the actors presented symptoms in the same order and presented the same body language^71^ strengthened the comparability of the characters. This is further supported by our finding that GPs did not differ in their perceived severity of the vignette characters’ conditions. Finally, having conducted a power analysis followed by both frequentist analyses as well as Bayesian estimation provides a statistically robust basis on which to draw the conclusions we have drawn.

## Conclusion

Our findings suggest that GPs may be influenced by patient background and gender when making clinical decisions regarding the management of MH. However, this is unlikely to be the result of differences in GPs' perceived clinical uncertainty. Future research should examine alternative explanations for the variation in GPs clinical decisions, such as their expectations of the types of perilous experiences refugees are likely to have had as well as their expectation of patients’ treatment preferences. Future research should furthermore pay careful attention to the validity of PTSD diagnoses, disparities in sick leave, and physical health medication given to refugee patients.

## Data Availability

Data are available in a public, open access repository. The data that support the findings of this study are openly available in OSF at osf.io/qexrj.
