# Supplementary material for: General practitioners’ management of depression symptoms in Somali refugee and Norwegian patients: a film vignette experiment
Source: BMJ Open. 2021 Dec 28;11(12):e055261. doi: 10.1136/bmjopen-2021-055261 (PMC8719221; doi:10.1136/bmjopen-2021-055261)
Supplement: Supplementary data [file bmjopen-2021-055261supp001.pdf]

Figure S1. CONSORT flow diagram of participant flow in the four groups.

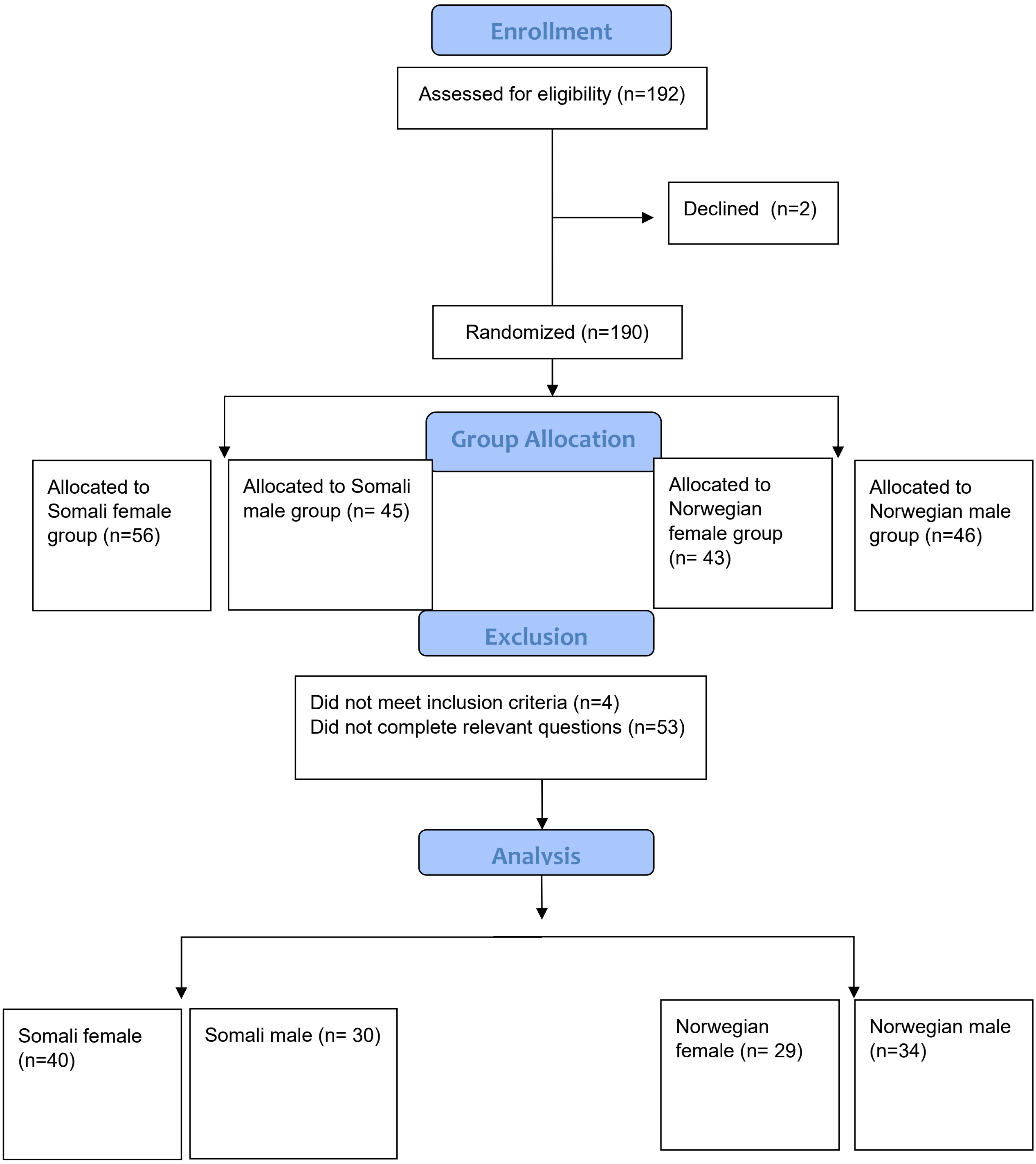

Figure S2. Vignette characters, female Norwegian (top left), female Somali (top right), male Norwegian (bottom left), male Somali (bottom right).

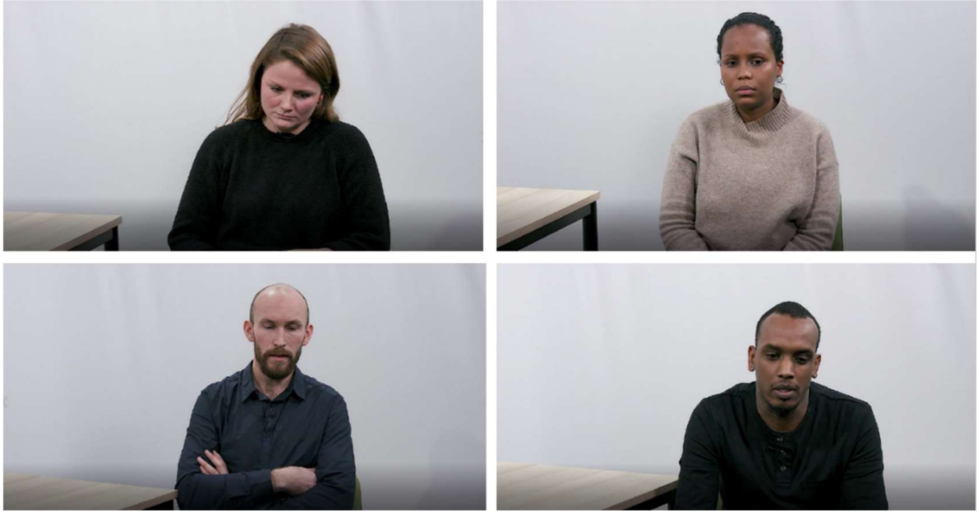

Box S1. Information given to GP participants before the online survey.

English translation
Imagine that you are a GP and have just arrived at the practice where you work. Your first patient is already waiting for you. You are currently substituting for a colleague of yours and have never met this patient. You have a few minutes to look through the patient's medical notes. You find the following information:<br><br>Name: Mari Berg/Emil Olsen/Hodan Osman/Abdi Warsame<br>Background: born and raised in Norway/refugee from Somalia<br>Time spent in Norway: NA/3 years<br>Age: 31<br>Married: Yes<br>Children: 2<br>Reason for today's consultation: Headache. The patient has previously been examined for headaches and no somatic cause was found.<br><br>You will now see a short film clip (4-5min) of a consultation with this patient. Then you will be asked to answer questions regarding how you would have assessed and treated this patient.

Table S1. Participant characteristics (N=133) by vignette character, including significance test for Pearson's Chi^2 comparison of groups.

	Vignette character (N%)				
**Participant characteristics**	**Mari** *N=29*	**Emil** *N=34*	**Abdi** *N=30*	**Hodan** *N=40*	***p***
Gender					0.864
Male	10 (34.5%)	10 (28.6%)	9 (30.0%)	10 (25.0%)	
Female	19 (65.5%)	24 (70.6%)	21 (70.0%)	30 (75.0%)	
Age					0.308
20-29	0 (0.0%)	2 (5.9%)	3 (10.0%)	4 (10.0%)	
30-39	16 (55.2%)	16 (45.1%)	17 (56.7%)	17 (42.5%)	
40-49	5 (17.2%)	9 (26.5%)	4 (13.3%)	11 (27.5%)	
50-59	2 (6.9%)	1 (2.9%)	2 (6.7%)	3 (7.5%)	
60+	6 (20.7%)	6 (17.6%)	4 (13.3%)	5(12.5%)	
Region of origin					0.714
Scandinavia	25 (86.2%)	32 (94.1%)	28 (93.3%)	34 (85.0%)	
Eastern Europe & Rest of Europe	2 (6.9%)	1 (2.9%)	0 (0.0%)	2 (5.0%)	
Africa & Asia	2 (6.9%)	1 (2.9%)	2 (6.7%)	4 (10.0%)	
Years of work experience					0.466
<10	17 (58.6%)	21 (61.8%)	22 (73.3%)	25 (62.5%)	
11-20	5 (17.2%)	6 (17.6%)	2 (6.7%)	8 (20.0%)	
21-30	1 (3.5%)	4 (11.8%)	2 (6.7%)	5 (12.5%)	
30+	6 (20.7%)	3 (8.8%)	4 (13.3%)	2 (5.0%)	
Region where education was undertaken					0.308
Scandinavia	20 (69.0%)	25 (73.5%)	27 (90.0%)	29 (72.5%)	
Eastern Europe	6 (20.7%)	7 (20.6%)	3 (10.0%)	10 (25.0%)	
Rest of Europe & Africa	3 (10.3%)	2 (5.9%)	0 (0.0%)	1 (2.5%)	
Distance from specialist health services (i.e. centrality)					0.471
<30min	17 (58.6%)	24 (70.6%)	23 (76.7%)	31 (77.5%)	
30-60	9 (31.0%)	5 (14.7%)	4 (13.3%)	4 (10.0%)	
60+	2 (6.9%)	3 (8.8%)	2 (6.7%)	2 (5.0%)	
I don't know	0 (0.0%)	0 (0.0%)	1 (3.3%)	0 (0.0%)	
I work at a specialist health service	1 (3.5%)	2 (5.9%)	0 (0.0%)	3 (7.5%)	
Course in migration and health taken during education*					0.097
Yes	12 (41.4%)	18 (52.9%)	15 (50.0%)	8 (20.0%)	
No	12 (41.4%)	10 (29.4%)	10 (33.3%)	20 (50.0%)	
I don't remember	5 (17.2%)	6 (17.6%)	5 (16.7%)	12 (30.0%)	
Course in migration and health taken after education*					0.136
Yes	9 (31.0%)	8 (23.5%)	6 (20.0%)	13 (32.5%)	
No	20 (69.0%)	23 (67.6%)	24 (80.0%)	22 (55.0%)	

Participant characteristics	Vignette character (N%)				
	Mari *N=29*	Emil *N=34*	Abdi *N=30*	Hodan *N=40*	*p*
I don't remember	0 (0.0%)	3 (8.8%)	0 (0.0%)	5 (12.5%)	
Need a course in migration and health*	15 (51.7%)	27 (79.4%)	21 (70.0%)	26 (65.0%)	0.132

*asked after presentation of the film vignette. NB categories with few participants were combined. Categories with 0 participants were dropped. This included 'Latin America', 'North America', and 'Oceania' for region of origin, 'Latin America', 'North America', 'Asia', and 'Oceania' for region of education, and '>50 years' for work experience.

Table S2. Table of frequentist 2x2 ANOVAs and interactions of participants' certainty about clinical decisions by sex and background of vignette character. Mean refers to GPs' average certainty score (0=not certain at all, 7=very certain).

Clinical decision	DF	Mean(SD)	Sum of Squares	Mean Squares	F Value	p-value
Diagnosis 1						
**Sex**	1		0.16	0.1573	0.079	0.779
Female		5.3(1.3)				
Male		5.3(1.5)				
**Background**	1		0.09	0.0931	0.047	0.829
Somali		5.3(1.4)				
Norwegian		5.3(1.4)				
**Sex*Background**	1		0.05	0.0517	0.026	0.872
Diagnosis 2						
**Sex**	1		0.02	0.0165	0.008	0.928
Female		5.1(1.5)				
Male		5.1(1.4)				
**Background**	1		0.01	0.0068	0.003	0.954
Somali		5.1(1.4)				
Norwegian		5.1(1.4)				
**Sex*Background**	1		0.02	0.0221	0.011	0.917
Diagnosis 3						
**Sex**	1		0.08	0.085	0.030	0.863
Female		4.6(1.7)				
Male		4.7(1.7)				
**Background**	1		2.15	2.152	0.766	0.384
Somali		4.5(1.6)				
Norwegian		4.8(1.8)				
**Sex*Background**	1		5.25	5.249	1.869	0.175
Assessment 1						
**Sex**	1		0.07	0.0667	0.043	0.837
Female		6.2(1.4)				
Male		6.2(1.1)				
**Background**	1		0.19	0.1938	0.124	0.726
Somali		6.2(1.3)				
Norwegian		6.2(1.2)				
**Sex*Background**	1		0.67	0.6673	0.426	0.515
Assessment 2						
**Sex**	1		0.25	0.2532	0.171	0.680
Female		5.9(1.3)				

Male		5.9(1.1)				
**Background**	1		1.87	1.8653	1.258	0.264
Somali		6.0(1.1)				
Norwegian		5.8(1.3)				
**Sex*Background**	1		0.03	0.0260	0.018	0.895
Assessment 3						
**Sex**	1		1.45	1.4529	0.683	0.411
Female		5.5(1.2)				
Male		5.3(1.7)				
**Background**	1		0.47	0.4704	0.221	0.639
Somali		5.3(1.5)				
Norwegian		5.4(1.4)				
**Sex*Background**	1		2.80	2.7992	1.315	0.254
Treatment 1						
**Sex**	1		0.54	0.536	0.424	0.516
Female		6.2(1.1)				
Male		6.1(1.2)				
**Background**	1		5.45	5.452	4.318	0.040
Somali		5.9(1.3)				
Norwegian		6.3(0.9)				
**Sex*Background**	1		0.44	0.443	0.351	0.555
Treatment 2						
**Sex**	1		1.66	1.659	0.929	0.337
Female		5.3(1.4)				
Male		5.5(1.3)				
**Background**	1		0.01	0.009	0.005	0.945
Somali		5.4(1.4)				
Norwegian		5.4(1.3)				
**Sex*Background**	1		4.25	4.248	2.379	0.126
Treatment 3						
**Sex**	1		4.21	4.209	2.040	0.158
Female		4.3(1.5)				
Male		4.8(1.5)				
**Background**	1		4.99	4.992	2.420	0.124
Somali		4.8(1.5)				
Norwegian		4.4(1.5)				
**Sex*Background**	1		4.26	4.257	2.064	0.155
